# Innovation and competition in advanced therapy medicinal products

**DOI:** 10.15252/emmm.201809992

**Published:** 2019-02-15

**Authors:** Enrique Seoane‐Vazquez, Vaishali Shukla, Rosa Rodriguez‐Monguio

**Affiliations:** ^1^ Chapman University School of Pharmacy Irvine CA USA; ^2^ Chapman University School of Pharmacy Irvine CA USA; ^3^ Director of the Medication Outcomes Center School of Pharmacy University of California San Francisco CA USA

**Keywords:** Genetics, Gene Therapy & Genetic Disease, Stem Cells

## Abstract

Advanced therapy medicinal products (ATMPs), including gene therapy, cell therapy and tissue engineering products, represent a paradigm shift in health care, but they are expensive. E. Seoane‐Vazquez, V. Shukla and R. Rodriguez‐Monguio discuss ATMPs prospects for the generics market.

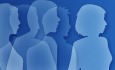

Advanced therapy medicinal products (ATMPs), including gene therapy, cell therapy, and tissue engineering products, represent a paradigm shift in health care as they have great potential for preventing and treating many diseases (Food and Drug Administration (FDA), 2013). By way of example, only 367 (8.0%) of the 4,603 rare diseases and conditions listed by the NIH Genetic and Rare Diseases Information Center had at least one FDA‐approved drug therapy in early 2018. An estimated 3,038 (66.0%) of those rare diseases and conditions are congenital and genetic diseases that could potentially be treated by gene therapy. There are already ATMPs under development to address these and many other unmet medical needs (FDA, 2013; MIT NEWDIGS FoCUS Project, 2017) and for the treatment of prevalent conditions, such as cardiovascular, neurologic, and metabolic diseases.

However, the high cost of ATMPs has given rise to concerns about the affordability of these breakthrough therapies for health care systems and patients. Furthermore, the projected increase of ATMP approvals in the upcoming decades will create a significant financial challenge for patients, insurance companies, and public health care schemes. During the past decades, the rise of generic markets for drugs and biologics has helped to drive down the costs for many drugs and other medicinal products; a similar market for generic versions of ATMP could therefore help to make these advanced treatments more affordable. Yet, there are several factors that may hinder a future competitive market for ATMPs and thereby affordability.

## ATMP authorizations and prices

In the USA and Europe, the regulation of ATMPs falls under the biologic licensing procedures of the FDA and the European Medicines Agency (EMA), respectively. Both agencies have implemented a regulatory framework to expedite the development and approval of ATMPs that address unmet medical needs or provide new therapies for serious or life‐threatening diseases. Both regulatory frameworks also enable the use of technical requirements adapted to the characteristics of each ATMP and explicitly enable companies and other developers to gain access to scientific and regulatory advice from the respective agency. Medical devices intended for use with a specific ATMP may be evaluated either as part of the ATMP or as stand‐alone devices. There are differences in the classification of ATMPs by the FDA and the EMA. The FDA classifies hematopoietic progenitor cell (HPC) cord blood products as an AMTP. The EMA considers that HPC cord blood products do not fit the definition of AMTP because they are not subject to substantial manipulation and are intended to be used for the same essential function in the recipient and the donor.

As of December 31, 2018, the FDA had authorized 16 ATMPs (11 cell therapies, including eight HPC cord blood products; four gene therapy products and one tissue engineering product), and the EMA had authorized 13 ATMPs (three cell therapies, six gene therapy products, and four tissue engineering products; Table 1). Six products (autologous cultured chondrocytes on a porcine collagen membrane‐specific marker proteins, axicabtagene ciloleucel, sipuleucel‐T, talimogene laherparepvec, tisagenlecleucel, and voretigene neparvovec) were authorized by both agencies. The FDA and EMA also granted orphan designation to 4 and 8 ATMPs, respectively. These products address several disease areas including progenitor cell transplantation, cancer, and cartilage defects. However, companies had also withdrawn 4 ATMPs authorized by the EMA from the market, citing commercial reasons for discontinuing their availability.

**Table 1:** FDA and EMA ATMP market authorizations and prices

Established name	Manufacturer	Agency	Authorization date	Market withdrawal	Price at market entry (US$)	Price type
Gene therapy						
Alipogene tiparvovec	UniQure	EMA	10/25/2012	10/28/2017	$1,206,751	Germany, Retail
Autologous CD34^+^ enriched cell fraction that contains CD34^+^ cells transduced with retroviral vector that encodes for the human adenosine deaminase (ADA) cDNA sequence from human hematopoietic stem/progenitor (CD34^+^) cells	GlaxoSmithKline	EMA	5/26/2016	Marketed	$738,223	UK, Retail excludes VAT
Axicabtagene ciloleucel	Gilead Sciences	EMA	8/23/2018	Marketed	NA	
		FDA	10/18/2017	Marketed	$373,000	US, Wholesale Acquisition Cost (WAC)
Talimogene laherparepvec	Amgen	EMA	12/16/2015	Marketed	$357,309	Germany, Retail
		FDA	10/27/2015	Marketed	$466,077	US, WAC
Tisagenlecleucel	Novartis	EMA	8/22/2018	Marketed	$441,538; $413,120	Germany, Retail; UK, Proposed by company
		FDA	8/30/2017	Marketed	$475,000	US, WAC
Voretigene neparvovec	Spark Therapeutics	EMA	11/23/2018	Marketed	NA	
		FDA	12/19/2017	Marketed	$850,000	US, WAC
Tissue‐engineered products						
Characterized viable autologous cartilage cells expanded *ex vivo* expressing specific marker proteins	TiGenix	EMA	11/16/2009	7/29/2016	$21,926	UK, Proposed by company
Autologous cultured chondrocytes on a porcine collagen membrane‐specific marker proteins	Vericel	EMA	6/27/2013	9/5/2014	$21,926	UK, Proposed by company
		FDA	12/13/2016	Marketed	$38,179	US, WAC
*Ex vivo* expanded autologous human corneal epithelial cells containing stem cells	Chiesi Farmaceutici	EMA	2/17/2015	Marketed	$93,432	UK, Retail excludes VAT
Spheroids of human autologous matrix‐associated chondrocytes	Don AG	EMA	7/10/2017	Marketed	$18,950	UK, Proposed by company
Cell therapy						
Allogeneic cultured keratinocytes and fibroblasts in bovine collagen	Organogenesis Incorporated	FDA	3/9/2012	Marketed	NA	
Allogeneic T cells genetically modified with a retroviral vector encoding for a truncated form of the human low‐affinity nerve growth factor receptor (ΔLNGFR) and the herpes simplex I virus thymidine kinase (HSV‐TK Mut2)	MolMed Spa	EMA	8/18/2016	Marketed	$814,780	Germany, Retail
Azficel‐T	Fibrocell Technologies	FDA	6/21/2011	Marketed	NA	
Darvadstrocel	Takeda Pharma	EMA	3/23/2018	Marketed	NA	
Hematopoietic progenitor cell cord blood	Cleveland Cord Blood Center	FDA	9/1/2016	Marketed	NA	
	SSM Cardinal Glennon Children's Medical Center	FDA	5/30/2013	Marketed	NA	
	Bloodworks	FDA	1/28/2016	Marketed	NA	
	Clinimmune Labs, University of Colorado Cord Blood Bank	FDA	5/24/2012	Marketed	NA	
	Duke University School of Medicine	FDA	10/4/2012	Marketed	NA	
	LifeSouth Community Blood Centers	FDA	6/13/2013	Marketed	NA	
	New York Blood Center	FDA	11/10/2011	Marketed	NA	
	MD Anderson Cord Blood Bank	FDA	6/21/2018	Marketed	NA	
Sipuleucel‐T	Dendreon	EMA	9/6/2013	5/6/2015	$110,920	Germany, Retail
		FDA	4/29/2010	Marketed	$141,005	US, WAC

ATMP, Advanced therapy medicinal product; EMA, European Medicines Agency; FDA, Food and Drug Administration.

The manufacturer price for an ATMP treatment ranges from US$18,950 for a tissue‐engineered product to US$1,206,751 for a gene therapy (Table 1). On average, prices are higher for gene therapy (US$357,309–US$1,206,751) than for cell therapy (US$110,920–US$814,780) and tissue‐engineered products (US$ $18,950–US$93,432). Yet, these prices do not include purchasing, inventory, and management costs that may significantly increase the overall treatment cost. By way of comparison, the treatment cost for the four ATMPs approved by both regulatory agencies was higher in the USA than in Europe: tisagenlecleucel costs 15.0% more in the United States than in Europe, sipuleucel‐T 27.1%, talimogene laherparepvec 30.4%, and autologous cultured chondrocytes 74.1%.

One major factor for the high cost of AMTPs is that these cater to only a small number of patients—often qualifying for orphan drug designation—and are used in personalized medicine (FDA, 2013). For example, the EMA authorized a gene therapy using autologous CD34^+^ cells to cure ADA‐SCID, a rare disease that affects between one in 200,000 and one in 1,000,000 children. This gene therapy is administered at a single specialist center in Italy. Other factors for the high prices of ATMPs include the current intellectual property regulation that limits competition and reimbursement mechanisms. European public health care systems and US payers usually cover the costs for most ATMPs, especially those without therapeutic alternatives.

While high prices may incentivize ATMP development, they limit accessibility and could even lead to market discontinuations for commercial reasons (Halioua‐Haubold *et al*, 2017). Market withdrawals are also related with the high cost associated with maintaining manufacturing capabilities, patient registries and risk management procedures, post‐marketing studies, development and validation of assays or regulatory reassessments, and other regulatory inspections.

## Market competition for generics

As ATMPs are costly for health insurance schemes and patients, competition will be essential for improving affordability and for these products to become mainstream medicine. The history of and experience with generic drugs, biosimilar products, and medical devices illustrates how increased market competition has benefited medical care during the past decades. In 1984, the Waxman Hatch Act (WHA) enabled the growth of the generic drug industry in the USA and elsewhere by establishing an abbreviated new drug application (ANDA) process for the review and approval of generic drugs. A generic drug company can use the safety and efficacy data of the reference drug, typically the original new drug application (NDA), to prepare an ANDA without the need to replicate costly clinical studies. Upon demonstration of chemical and biological equivalence during the ANDA review, the FDA determines the therapeutic equivalence and interchangeability of the generic drug. The WHA also created a process for generic companies to challenge the validity of brand drug patents and allowed companies to use patented drugs to prepare an ANDA. The ANDA process was eventually adapted by regulatory agencies in Europe and by the EMA at its inception in 1995.

The FDA and the EMA have also established routes for the review and approval of biosimilar alternatives to branded biologic products. The process is more stringent and costly than the process for generic drugs, partially because it is not possible to exactly replicate complex biologic products and demonstrate bioequivalence. To get approved, a biosimilar product must have the same route of administration, dosage form, and strength as the reference product. Additionally, the application must demonstrate that the product is highly similar to the reference product based on data from analytical, animal, and clinical studies. A biosimilar product that meets these standards is considered interchangeable with the reference biologic.

The generic and biosimilar markets are examples of how market competition can reduce prices, improve affordability, and expand access to therapies. For example, aripiprazole, an atypical antipsychotic, and imatinib mesylate, an antineoplastic agent, experienced important reductions in cost after generic entry. In February 2018, two and a half years after generic entry, the community pharmacy National Average Drug Acquisition Cost (NADAC) collected by the Centers for Medicare and Medicaid Services for 30 day supply of aripiprazole 10MG tablet was 78 times lower for the generic than for the brand version of the drug ($11 and $857, respectively). In the case of imatinib mesylate tablet 400MG, the 30‐day supply NDAC price was 18.5 times lower for the generic than for the brand ($529 vs. $9,808) version of the drug in October 2018, two and a half years after generic entry. Savings associated with the use of biosimilars can also be substantial. In October 2018, the manufacturer average sales price reported by CMS for a day of treatment of filgrastim (leukocyte growth factor) was 43% lower for the biosimilar than the reference biologic product ($203 vs. $355, respectively).

The acceptance of low‐cost alternative generics and biosimilar products has been facilitated by significant savings. In 2016, generic drugs represented 90% of the prescriptions and 26% of the drug expenditures in the USA (IQVIA Institute for Human Data Science, 2017). Nevertheless, the number of biosimilars authorized in the USA remains limited. The FDA approved the first biosimilar product (filgrastim, a recombinant‐DNA form of granulocyte colony‐stimulating factor used in cancer therapy) in March 2015. In the fourth quarter of 2016, two alternative products to filgrastim captured 30% of the market (Mulcahy *et al*, 2017). By March 2018, the FDA had approved nine biosimilar products for six different biologics.

In the EU, the market share of generic drugs varies by country from 11 to 81% of the drug units and 6 to 36% of the expenditures (OECD, 2017). The EMA approved the first biosimilar (somatotropin) in April 2006; by March 2018, the EMA had approved 39 biosimilar products corresponding to 15 different biologic products. The EU market share of biosimilars as a percent of sales also varies by product. For example, biosimilar market sales represented 4% of the reference insulins and 88% of granulocyte colony‐stimulating factors in 2016 (QuintilesIMS, 2017).

In the case of medical devices, premarket approval applies to devices that support or sustain human life, are of significant importance in preventing impairment of human health, or present a potential, unreasonable risk of illness or injury. Competition is limited for devices that are subject to premarket authorization processes. The pre‐approval process requires valid scientific evidence that the device is safe and effective for its intended use and may require clinical trials. A device can be exempted from premarket regulatory requirements if it is considered safe, effective, and substantially equivalent to a legally marketed device and enters the market after notification to the regulatory organizations (FDA in the USA and notified bodies in the EU). Overall, the bioequivalence of generic drugs (Carpenter & Tobbell, 2011), the similarity of biosimilars, and the substantial equivalence of medical devices are the main factors allowing for product interchangeability and therefore efficient market competition.

## Demonstrating biosimilarity for ATMPs

While the generics market for drugs, medical devices, and some biologics has been driving down prices and improving affordability and access to medicinal products, the challenge to establish a similar market of generics makers of ATMPs is considerably greater. A first barrier would be the current regulatory scheme itself. ATMP is a complex and dynamic regulatory area compared with the now well‐established regulation of drugs, biologics, and medical devices. Products classified under the ATMP umbrella can be very diverse, and current regulations are product‐specific and focus on incentivizing innovation. It would first require a stable and well‐defined ATMP regulatory framework that sets reference standards and criteria for approval, along with implementing regulatory pathways for ATMP biosimilar review and authorization. The demonstration of biosimilarity for ATMPs is also challenging because those products are often complex active substances, patient‐specific (autologous), or require careful matching of donor and recipient (allogeneic). Moreover, only few validated biomarkers for establishing biosimilarity have been identified. Regulatory systems must address the complexity of ATMPs and the difficulty of comparing outcomes and demonstrating biosimilarity of highly individualized therapies to ensure clinical equivalence.

The development of biosimilar ATMPs will also depend on the cost and barriers to access the innovator product, which is required to perform comparative studies. In addition, biosimilar ATMP clinical studies—if required—will be challenging and costly and face the same difficulties in enrolling sufficient number of patients as the original product. The success of a ATMP biosimilar industry will also require the development of technologies to enable large‐scale, reproducible, and cost‐effective manufacturing of high‐quality products (Dwarshuis *et al*, 2017).

Lastly, while the current regulatory framework for ATMPs focuses on providing companies with incentives for innovation, it is not necessarily supporting competition. Aligning the necessary incentives for research and development with health care budgetary constraints is an important challenge for policymakers and regulatory agencies (Rodriguez‐Monguio *et al*, 2017). Yet, the complexities of the regulatory framework, clinical effectiveness, and safety, along with the economic and ethical issues of ATMP innovation, access, and affordability, have not been sufficiently discussed so far.

## Conclusions

To date, only a few ATMPs have been approved by FDA and EMA. However, many more therapies are under preclinical/clinical development and are expected to reach the market in the foreseeable future. The high cost of ATMP limits affordability for public and private payers and reduces patient access to treatment for what are often life‐threatening conditions and diseases. Current regulatory and policy initiatives focus on encouraging innovation and expediting review of ATMPs rather than on enabling market competition and thereby ensuring affordability and availability of these new therapies. A greater market and ensuing competition for ATMP biosimilars will be limited by the complexity of ATMPs, fast technological evolution, difficulties in demonstrating clinical equivalence, the high cost of development and manufacturing, and the lack of a well‐defined regulatory framework for review and authorization of biosimilar ATMPs.
